# Supplementary material for: The Antioxidant and Anti-Aging Effects of Acetylated Mycelia Polysaccharides from Pleurotus djamor
Source: Molecules. 2019 Jul 24;24(15):2698. doi: 10.3390/molecules24152698 (PMC6696436; doi:10.3390/molecules24152698)
Supplement: Supplementary file 1 [file molecules-24-02698-s001.docx]

Supplementary Files 1

**Single-Factor test:**

DS for a series of material liquid ratios.

| **Reaction time (h)** | **DS** | **SD** |
| --- | --- | --- |
| 6:1 | 0.171 | 0.015 |
| 3:1 | 0.212 | 0.021 |
| 1:1 | 0.384 | 0.017 |
| 1:3 | 0.495 | 0.023 |
| 1:6 | 0.433 | 0.019 |

DS for a series of reaction times.

| **Reaction time (h)** | **DS** | **SD** |
| --- | --- | --- |
| 12 | 0.121 | 0.017 |
| 24 | 0.393 | 0.023 |
| 36 | 0.456 | 0.02 |
| 48 | 0.518 | 0.015 |
| 60 | 0.51 | 0.023 |

DS for a series of reaction temperatures.

| **Reaction temperature (°C)** | **DS** | **SD** |
| --- | --- | --- |
| 23 | 0.193 | 0.023 |
| 30 | 0.475 | 0.019 |
| 37 | 0.52 | 0.015 |
| 44 | 0.429 | 0.017 |
| 51 | 0.244 | 0.018 |

DS for a series of amount of acetic anhydride added.

| **Amount of acetic anhydride added (mL)** | **DS** | **SD** |
| --- | --- | --- |
| 1 | 0.132 | 0.03 |
| 2 | 0.334 | 0.017 |
| 3 | 0.416 | 0.02 |
| 4 | 0.492 | 0.016 |
| 5 | 0.431 | 0.015 |

Response Surface Optimization

Response surface test factor level and number of degree of acetyl substitution in AMPS.

| **Variable** | Code  Uncoded Coded | | −1 | Levels  0 | 1 |
| --- | --- | --- | --- | --- | --- |
| **Reaction time（h）** | X_1_ | A | 36 | 48 | 60 |
| Reaction temperature(^◦^C) | X_2_ | B | 30 | 37 | 44 |
| Amount of acetic anhydride added（mL） | X_3_ | C | 3 | 4 | 5 |

Response surface experimental design of degree of acetyl substitution in AMPS.

|  | **A** | **B** | **C** | DS | |
| --- | --- | --- | --- | --- | --- |
|  |  |  |  | Actual Value Predicted Value | |
| 1 | –1 | –1 | 0 | 0.43 ± 0.04 | 0.43 |
| 2 | 1 | –1 | 0 | 0.44 ± 0.05 | 0.45 |
| 3 | –1 | 1 | 0 | 0.4 ± 0.03 | 0.39 |
| 4 | 1 | 1 | 0 | 0.45 ± 0.04 | 0.45 |
| 5 | –1 | 0 | –1 | 0.49 ± 0.02 | 0.49 |
| 6 | 1 | 0 | –1 | 0.52 ± 0.04 | 0.51 |
| 7 | –1 | 0 | 1 | 0.43 ± 0.03 | 0.44 |
| 8 | 1 | 0 | 1 | 0.5 ± 0.04 | 0.50 |
| 9 | 0 | –1 | –1 | 0.43 ± 0.05 | 0.43 |
| 10 | 0 | 1 | –1 | 0.44 ± 0.04 | 0.45 |
| 11 | 0 | –1 | 1 | 0.44 ± 0.03 | 0.43 |
| 12 | 0 | 1 | 1 | 0.39 ± 0.04 | 0.39 |
| 13 | 0 | 0 | 0 | 0.54 ± 0.05 | 0.54 |
| 14 | 0 | 0 | 0 | 0.53 ± 0.03 | 0.54 |
| 15 | 0 | 0 | 0 | 0.53 ± 0.04 | 0.54 |
| 16 | 0 | 0 | 0 | 0.54 ± 0.03 | 0.54 |
| 17 | 0 | 0 | 0 | 0.52 ± 0.04 | 0.54 |

The predicted value of DS can be calculated by the following formula:

DS = 0.54 + 0.02 × A – 7.5 × 10^-3^ × B – 0.015 × C + 0.01 × A × B + 0.01 × A × C – 0.015 × B × C – 0.025 × A^2^ – 0.085 × B^2^ – 0.03 × C^2^.

ANOVA analysis of degree of acetyl substitution in AMPS.

| **Source** | **Sum of Squares** | **df** | **Mean Square** | ***F*-value** | **P-value** |
| --- | --- | --- | --- | --- | --- |
| Model | 3.200 × 10^-3^ | 9 | 5.181× 10^-3^ | 79.05 | <0.0001 |
| A | 4.500 × 10^-4^ | 1 | 3.200 × 10^-3^ | 48.82 | 0.0002 |
| B | 1.800 × 10^-3^ | 1 | 4.500 × 10^-4^ | 6.87 | 0.0344 |
| C | 4.000 × 10^-4^ | 1 | 1.800 × 10^-3^ | 27.46 | 0.0012 |
| AB | 4.000 × 10^-4^ | 1 | 4.000 × 10^-4^ | 6.10 | 0.0428 |
| AC | 9.000 × 10^-4^ | 1 | 4.000 × 10^-4^ | 6.10 | 0.0428 |
| BC | 2.611× 10^-3^ | 1 | 9.000 × 10^-4^ | 13.73 | 0.0076 |
| A^2^ | 0.030 | 1 | 2.611 × 10^-3^ | 39.83 | 0.0004 |
| B^2^ | 3.764 × 10^-3^ | 1 | 0.030 | 463.05 | <0.0001 |
| C^2^ | 3.200 × 10^-3^ | 1 | 3.764 × 10^-3^ | 57.43 | 0.0001 |
| Lack of Fit | 4.500 × 10^-4^ | 3 | 1.500 × 10^-4^ | 68.18 | 0.0580 |
